# Ultrahigh-pressure supercritical fluid extraction and chromatography of *Moringa oleifera* and *Moringa peregrina* seed lipids

**DOI:** 10.1007/s00216-019-01850-x

**Published:** 2019-05-04

**Authors:** Yannick Nuapia Belo, Said Al-Hamimi, Luke Chimuka, Charlotta Turner

**Affiliations:** 1 Molecular Sciences Institute, School of Chemistry, University of the Witwatersrand, Private Bag X3, Johannesburg, 2050 South Africa; 2 Department of Chemistry, Centre for Analysis and Synthesis, Lund University, P.O. Box 124, 22100 Lund, Sweden

**Keywords:** Ultrahigh-pressure supercritical fluid extraction, *Moringa oleifera*, *Moringa peregrina*, Lipid profiling, Sonication, Phospholipids, SFE

## Abstract

An ultrahigh-pressure supercritical fluid extraction method was optimized and applied to extract seed oil lipids from two moringa species, namely *Moringa oleifera* (MO) and *Moringa peregrina* (MP). A full-factorial design was used to investigate the direct and interaction influence of pressure and temperature in the range of 40 to 80 MPa and 40 to 70 °C, respectively, on the extracted amount of oil from crushed seeds. The results revealed that pressure has a significant positive influence on the extracted amount of oil. The best extraction condition using neat CO_2_ was found at 80 MPa and 57 °C, yielding 396 ± 23 and 529 ± 26 mg oil per gram of seeds for MO and MP, respectively. An extraction kinetics study revealed a mainly solubility-controlled extraction of oil, and 28 g of CO_2_ was required to extract 400 mg of oil per gram of seeds of MO using the developed method. Addition of ethanol to the sample prior to the extraction increased the proportion of extractable polar lipids as well as the total amount of extracted oil. The developed method increased the extracted amount of oil twofold compared to a reference method based on solvent sonication. The obtained oil consisted mainly of glycerolipids, sterol esters, and phospholipids. Phospholipids, campesterol, and stigmasterol ester concentrations were found to be higher in MO while cholesterol ester was more abundant in MP.

**Electronic supplementary material:**

The online version of this article (10.1007/s00216-019-01850-x) contains supplementary material, which is available to authorized users.

## Introduction

Moringa plants belong to the family of the Moringaceae. The trees are widely distributed among tropical and sub-tropical countries. The moringa family includes 13 species, of which *Moringa oleifera* (MO) is the most widely cultivated species in Africa and India. It is also known as the “drumstick” or “horseradish” tree. *Moringa peregrina* (MP), on the other hand, grows naturally, covering a wide range from the Dead Sea area sporadically along the Red Sea coasts to northern Somalia and around the Arabian Peninsula to the mouth of the Persian Gulf [1]. Due to the medicinal and nutritional values of moringa, every part of the tree including leaves, stems, flowers, and seeds has been utilized as a source of various therapeutic bioactive compounds in medicinal and pharmacological products. The leaves are an excellent source of flavonoids, phenolics, saponins, proteins, and vitamins [2]. The seed can contain up to 34 to 60 wt% of oil called “ben oil.” It is rich in behenic, oleic, stearic, and palmitic acids. Moringa seeds contain a higher amount of polyunsaturated fatty acids compared to palm, soybean, and sunflower oil [1]. The quality of moringa seed oil is similar to that of olive oil as reported in the literature [3, 4], i.e., being an excellent source of unsaturated fatty acid, vitamin A, and vitamin E. In addition, moringa seed oil has antiseptic, antimicrobial, and anti-inflammatory activities [3]. These exceptional nutritional and medicinal values of the oil lead to their use in the cosmetic, food, and pharmaceutical industries.

The quality of MO oil has received the interest of many investigators to develop novel technologies for efficient extraction of edible oil. Pressurized liquid extraction (PLE) [5], ultrasound- [6] and microwave-assisted extraction [7], and supercritical fluid extraction (SFE) [8] are examples of such technologies. The drawback of these techniques is that they require a large amount of organic solvents and some of them are toxic. In the case of SFE, a co-solvent is essential to enhance the solubility of more polar lipids. In this study, we show the benefit of using ultrahigh-pressure supercritical fluid extraction (UHPSFE) to extract polar lipid with a minimum amount of organic solvents.

Extraction using supercritical carbon dioxide (ScCO_2_) as a solvent offers several benefits such as low toxicity, non-flammability, high diffusivity, gas-like viscosity, and liquid-like density [9]. In addition, its near-ambient critical temperature makes it suitable for the extraction of thermolabile high-value-added products without risking degradation [10]. Solubility of a compound in ScCO_2_ is generally suggested to be controlled by two parameters—the vapor pressure of the compound and its interactions with the ScCO_2_ [11]. These two parameters in turn are influenced by temperature and pressure. Therefore, temperature and pressure are principal factors that control the extraction process in SFE in terms of speed, efficiency, and selectivity. Temperature in SFE has contradictory effects depending on the operational range and type of analytes. For example, a higher temperature enhances mass transfer properties and increases vapor pressure of the analytes, which leads to a higher extraction efficiency. On the other hand, a higher temperature decreases the density of the ScCO_2_ which lowers the solvation power [11], an effect that is more pronounced at lower pressures. Hence, if a relatively high temperature is desired, e.g., over 60 °C, a quite high pressure is needed to achieve a high solvent density. For instance, increasing the pressure at 60 °C from 20 to 80 MPa increases the density from 0.724 to 1.02 g/mL. The increase in the density is associated with an increase in solubility, especially for oils and lipids.

Most commercial analytical-scale SFE equipment use a maximum pressure of 60 MPa, even though larger-scale engineering studies have shown that pressures over 60 MPa, often called ultrahigh-pressure SFE, inherently enable enhanced oil extractability, altered selectivity, and faster extractions. For instance, ultrahigh-pressure SFE has been used in the defatting of food ingredients like almond and cocoa powder [12]. Pressure between 60 and 85 MPa showed the best results to extract the maximum amount of oil from coriander seeds [12]. Recently, extraction at pressures up to 130 MPa was achieved to obtain apple seed oil [13]. The high pressure increased the extracted oil amount from 10 to 190 mg/g dry apple seeds when comparing 30 and 130 MPa. However, none of these studies were conducted based on the analytical chemistry approach, aiming for quantifiable amounts and method validation in terms of trueness and precision.

SFE has been applied at moderate pressures to extract oil from moringa seeds [8, 14, 15]. The impact of extraction pressure and temperature on the oil yield and quality was investigated in the range of 20 to 50 MPa and between 40 and 100 °C, respectively. As in most studies about SFE of lipids, pressure turned out to be a significant factor impacting the extractable amount. In these studies, the composition of the oil was evaluated through analysis of fatty acids by gas chromatography (GC) with flame ionization detector (FID) or mass spectrometry (MS) after hydrolysis and derivatization. Such analysis does not provide full information whether the fatty acids originally present as free fatty acids or attached to glycerolipids, phospholipids, or glycolipids.

To the best of our knowledge, there are no studies implementing high-pressure (> 60 MPa) ScCO_2_ for the quantitative extraction of oil from moringa species seeds. Moreover, there are no comprehensive lipid profiling studies of the oil extracted from moringa seeds. Hence, this study aims to develop an extraction method of oil from moringa seeds based on ultrahigh-pressure supercritical fluid extraction (UHPSFE) using a design of experiment (DoE) approach. Furthermore, we aim to conduct a comprehensive lipid profiling analysis of the obtained oil using ultrahigh-performance supercritical fluid chromatography coupled to high-resolution mass spectrometry (UHPSFC/QTOF-MS).

## Materials and methods

### Chemicals and materials

Ultrapure CO_2_ (99.999%) was provided by Air Products (Amsterdam, Netherlands). LC–MS-grade methanol was purchased from Scharlau (Barcelona, Spain). Heptane (99.5%) was purchased from Alfa Aesar (Karlsruhe, Germany). Ethanol 99.7% and HPLC-grade chloroform were from VWR (VWR Chemicals, France). MS-grade ammonium formate was purchased from Sigma-Aldrich (St. Louis, MO). Water was purified using a Milli-Q purification system (Millipore, Billerica, MA).

### Plant samples

Two species of moringa were subjected to extraction and analysis: *Moringa oleifera* from South Africa and *Moringa peregrina* from Oman. Dried *Moringa peregrina* seeds were collected from the Al-Dhakhlia region in Oman in June 2017 while dried *Moringa oleifera* seeds were collected from Limpopo province, South Africa, in September 2017. Prior to processing, peeled seeds were dried at 50 °C for 8 h and the water content was found to be 3.5–4.6% weight. The dried samples were crushed to powder and kept in a sealed container until extraction.

### Extraction with UHPSFE

A home-built extraction system was assembled consisting of an ISCO 65D syringe-pump (Teledyne Isco, Thousand Oaks, CA), a GC oven in which the extraction vessel was placed, and a needle valve connected to a restrictor (a long stainless steel tube with an internal diameter of 0.0036 in.) to control the flow rate. The extractions were started by weighing and loading 1 g of seed powder into a 5-mL stainless steel extraction vessel and mixing with 4 g of 3-mm-ID glass beads. The extraction vessel was fitted with metallic filters (0.45 μm pore size) at the inlet and the outlet. The extractions were carried out in continuous-flow mode using flow rates ranging between 2.2 and 2.8 mL/min controlled manually by the needle valve. The extracts were collected in pre-weighed glass tubes to record the extracted weight. The system was flushed with a CO_2_/n-heptane fluid mixture after each run for cleaning purposes. The extracted oil samples were kept in a freezer at − 20 °C. Thirty milligrams of the oil was re-dissolved in 1 mL of a mixture, n-heptane:isopropanol (1:1, *v*:*v*), for chromatographic analysis.

### Design of experiment

The effects of pressure (P) and temperature (T) on the extraction yield of oil were evaluated using a response surface methodology. A full-factorial design comprising 11 experiments with three center point replicates was created in MODDE 10.1 (Sartorius Stedim Biotech, Malmö, Sweden) to investigate the influence of temperature (40–70 °C) and pressure (40–80 MPa) on the extracted amount of oil. The range of the factors was chosen based on the limits of the instrument and extraction vessel material specifications. During the optimization process, the flow rate was kept in the range of 2.2–2.8 mL/min while the total solvent extraction volume was fixed to 40 mL per extraction. The volume was fixed due to the low capacity of the Isco pump which is 65 mL. Multiple linear regression (MLR) was used to calculate the fitting model and response surface. The adequacy of the models was evaluated by the *R*^2^ and *Q*^2^ values (where *R*^2^ shows the model fit and *Q*^2^ shows an estimate of the future prediction precision), predicted vs. observed plot, and coefficient plots. The optimum processing conditions were obtained by using graphical and numerical analysis based on the criteria of the desirability function and the response surface plots.

### Extraction with sonication

Ultrasonic solvent extractions were performed according to the procedure described by Xhaxhiu and Wenclawiak with some modification [16]. Briefly, 2 g of crushed seed was dispersed into a 100-mL conical flask filled with 25 mL pure n-heptane. The samples were treated in a sonication water bath for 20 min at 60 °C. The extract was collected and the residue was subjected to extraction with fresh solvent. This step was repeated twice. The extracts were combined and filtered through filter papers with 0.45 μm pores. The solvent was removed using a rotary evaporator, and the extracted amounts were measured gravimetrically. Thirty milligrams of the oil was re-dissolved in 1 mL of the mixture n-heptane:isopropanol (1:1, *v*:*v*) for chromatographic analysis.

### Lipid profiling by UHPSFC

Samples were analyzed by ultrahigh-performance supercritical fluid chromatography (UHPSFC) according to our previous method [17]. Two microliters of reconstituted oil was injected in Acquity UPC^2^ (Waters, Milford, MA) equipped with an Acquity Diol UPC^2^ column (100 mm × 3 mm, 1.7 μm, Waters). The flow rate and column temperature were set at 1.5 mL/min and 48 °C, respectively. An active back pressure regulator (ABPR) was set at 131 bar to control the pressure at the outlet of the column. Methanol containing 20 mM of ammonium formate was used as a modifier, and the gradient of the modifier added to the CO_2_ was as follows: 0 min, 1%; 3 min, 4%; 4 min, 19%; 8 min, 45%; 11 min, 45%; 12 min, 1%; and 14 min, 1%. The injector needle was washed with methanol after each injection.

The Acquity UPC^2^ was connected to a Xevo 2G QTOF-MS (Waters). Two T-pieces (Waters) were used for backpressure control and infusion of methanol (0.2 mL/min) as a makeup liquid. The MS was operated in positive and negative electrospray ionization (ESI) modes with a scan range of *m*/*z* 80–1200. In positive ESI mode, the capillary voltage was 3.2 kV, the sampling cone voltage 34 V, the source temperature 120 °C, the drying gas temperature 420 °C, the cone gas flow 40 L/h, and the drying gas flow 690 L/h. In negative ESI mode, the capillary voltage was 2.6 kV, the sampling cone voltage 42 V, the source temperature 120 °C, the drying gas temperature 390 °C, the cone gas flow 40 L/h, and the drying gas flow 740 L/h. A collision energy ramp between 15 and 55 eV was used for MS^E^, targeted MS/MS, and data-dependent MS/MS in both positive and negative ESI. Data was acquired using MassLynx v4.1 (Waters Technology).

### Data analysis

Raw data (*m*/*z*) generated by UHPSFC were processed using the open-source software package MZmine 2.28 (http://mzmine.sourceforge.net/) [18]. Data were generated by targeted peak detection with a *m*/*z* tolerance of 0.5 mDa or 5 ppm and a retention time tolerance of 0.2 min, respectively. Data were visualized by principal component analysis (PCA) calculated in SIMCA-P+ 12.0.1 (Sartorius Stedim Biotech, Malmö, Sweden). Data were compared and analyzed statistically using ANOVA with *t* test, and *p* value < 0.05 is considered significant.

## Results and discussion

### Optimization of the extraction process

Pressure and temperature were selected for optimization because they are the main factors that influence the solubility of lipids in ScCO_2_, and thereby also the extraction efficiency. The extracted amounts of oil obtained from MO at different experiments of the design are presented in Table 1. The model was created and calculated by fitting using multiple linear regression.

**Table 1:** Experimental conditions and the corresponding amount of extracted oil from MO seeds

Exp. no.	Run order	Pressure (MPa)	Temperature (°C)	Extracted amount of oil (mg)
1	9	40	40	238
2	3	40	70	270
3	7	80	40	330
4	1	80	70	359
5	5	60	40	299
6	6	60	70	317
7	11	40	55	296
8	10	80	55	396
9	2	60	55	338
10	4	60	55	320
11	8	60	55	310

**Fig. 1:**
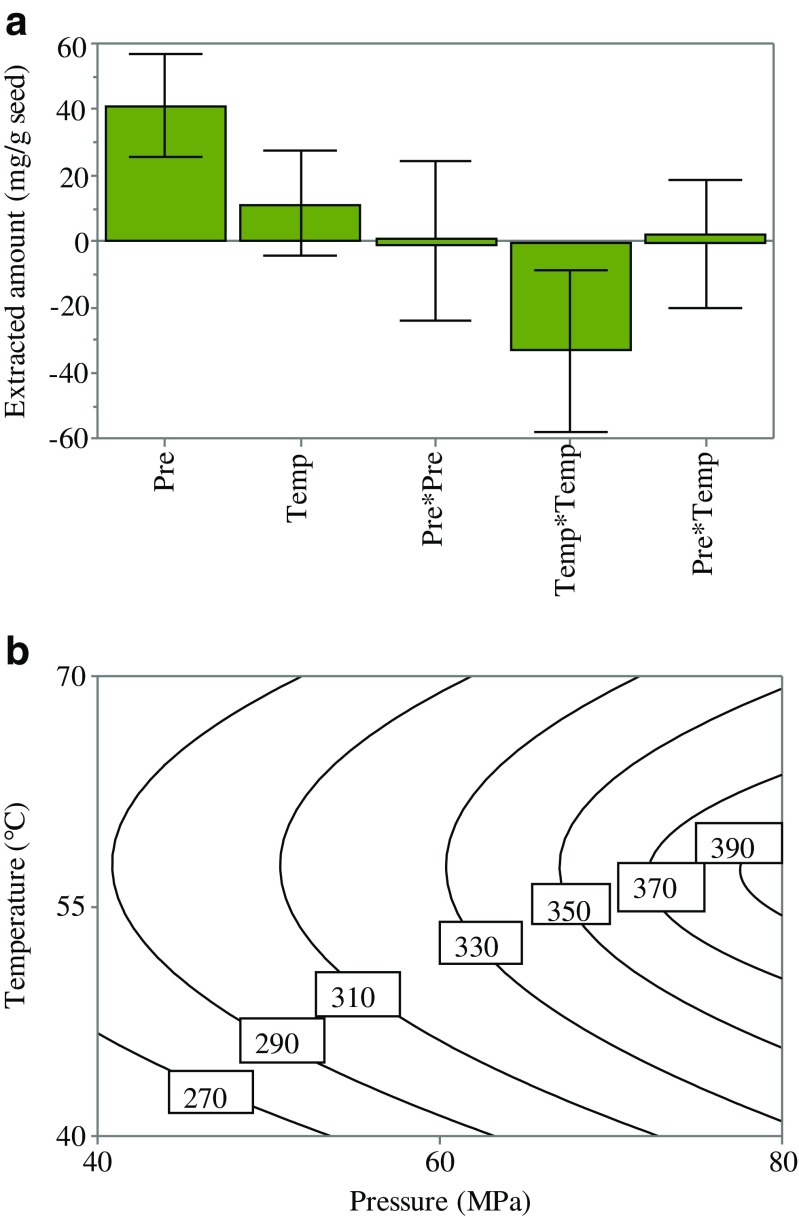
The plots obtained from the DoE model. **a** The coefficient plot of the examined variables and their influence on the extracted amount of oil from MO seeds. **b** The contour plot showing the extracted amount of oil (mg/g seed) at different settings of temperature and pressure

The fitted model showed a total explained variance of 94% (*R*^2^ = 0.94) and a cross-validated predictability of 85% (*Q*^2^ = 0.85), where *R*^2^ shows the model fit and *Q*^2^ shows an estimation of the future prediction and precision (see Electronic Supplementary Material (ESM) Fig. S1A). The linearity of the predicted versus observed values plot (see ESM Fig. S1B) underlined the validity of the model and its capability to predict the best condition of the extraction within the range of the design. The coefficients plot (Fig. 1a) reveals that pressure has a significant positive influence on the extracted amount of the oil with a *p* value of 0.001, whereas the interaction effect of temperature*temperature has the opposite effect.

The response surface represented by a counter plot in Fig. 1b shows the direct and the interaction impact of pressure and temperature on the extracted amount. Increasing the pressure from 40 to 80 MPa at 55 °C raised the extracted amount from 285 to about 395 mg. This is probably due to the increment in density of the fluid (from 0.91 to 1.03 g/mL). At a high density, the space between CO_2_ molecules diminishes and that enhances the interaction between oil and CO_2_, leading to a greater oil solubility in ScCO_2_ [19, 20]. In addition, a higher pressure may lead to greater rupturing of cell walls and more solvent penetration [21]. Nguyen et al. found that the solubility of MO oil increased by 3.4 times as the pressure of ScCO_2_ increased from 20 to 30 MPa at a constant temperature [22]. Rai et al. also found pressure to be the most significant parameter influencing oil yield from MO seeds using SFE [8].

Increasing the temperature from 40 to about 55 °C also increased the yield (Fig. 1b). This can be explained by the enhancement of mass transfer due to a decrease in solvent viscosity. Temperature influences positively through the increasing vapor pressure of the analytes, which might increase the extracted amount [11]. However, a further increase in temperature has a contradictory effect on the extracted amount, which might be explained by the reduction in the solvent’s density. It is clear that the optimal condition is found at the highest value of pressure (80 MPa) and the center point of temperature of 55 °C. For a systematic optimization, a desirability function was used to find the setting of pressure and temperature where the maximum extracted amount can be found. Accordingly, the best condition was found at 57 °C and 80 MPa. The predicted value of the yield at the best condition was 389 mg. To validate the model, extraction was repeated at the best condition and was found to be 396 ± 23 mg (*n* = 3) which fits well to the predicted value.

**Fig. 2:**
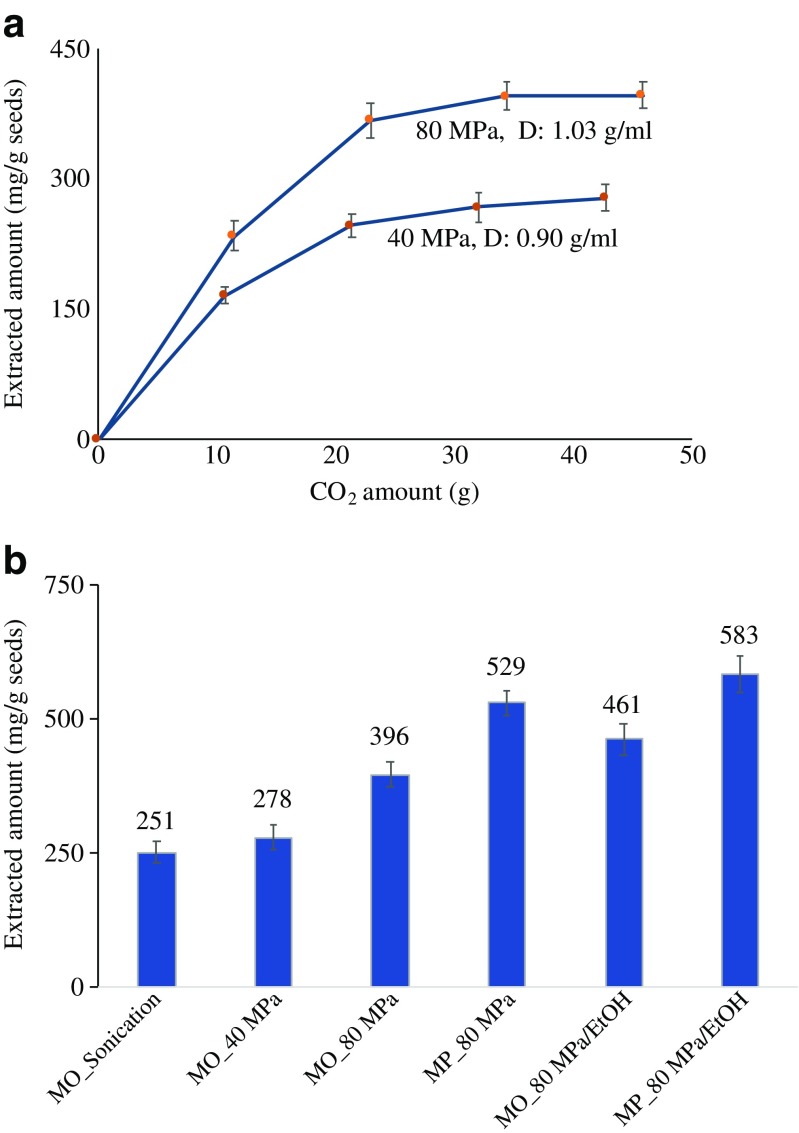
**a** Extracted amount of oil (mg) vs. solvent amount (g) from MO seeds using neat ScCO_2_ at 40 and 80 MPa, respectively, and a temperature of 57 °C. **b** Extracted amount of oil from MO and MP seeds using sonication and neat ScCO_2_ at 40 and 80 MPa, and with modifier at 80 MPa and 57 °C

The effect of the volume of the extraction solvent was examined for two extraction cycles (the pump was refilled between them), each cycle with 40 mL at the optimal extraction condition (57 °C and 80 MPa). The results showed that an additional 40 mL extraction cycle did not increase the extracted amount, which indicated that the extraction was completed in the first cycle. For verification, an extraction kinetics study was performed by collecting fractions each 10 mL of used CO_2_. Figure 2a shows extraction curves obtained at two different pressures, 40 and 80 MPa, at a fixed temperature of 57 °C and an average flow rate of 2.2 mL/min. The volume of CO_2_ (mL) is converted to mass (g) by calculating the density of CO_2_ at 5 °C (chiller temperature) and pressures of 40 and 80 MPa using an online software (www.peacesoftware.de). At 80 MPa, the total extracted amount is about 400 mg/g seeds compared to 278 mg/g seeds at 40 MPa. The extraction kinetics curve in Fig. 2a reveals that more than 50% of the extractable oil is recovered after extracting the sample with 10 g of CO_2_ at 80 MPa. In addition, the extraction process was completed after using about 28 g of CO_2_ at 80 MPa as indicated by the plateau curve (Fig. 2a). In comparison, extracting the sample with 40 g of CO_2_ at 40 MPa is not adequate to complete the extraction of the oil as shown in Fig. 2a in the lower curve. The curve of 40 MPa indicates that the extracted amount increases continuously as more solvent is used. Note that the flow rate was not optimized in this study since it was manually controlled which inherently introduces errors.

Ideally, a modifier is added as a co-solvent with ScCO_2_ throughout the extraction process. Due to the lack of appropriate equipment, the impact of the presence of the modifier was studied by adding 1 mL of ethanol into the extraction vessel before the extraction. Addition of ethanol combined with 5 min static extraction was followed by dynamic extraction with 28 g ScCO_2_ at 80 MPa and a flow rate around 2 mL/min. The extracted amount with the modifier was 461 ± 26 mg per gram of MO seeds. The limitation of ScCO_2_ is that its polarity is too low to obtain efficient or complete extractions, because most analytes lack sufficient solubility or the solvent has a poor ability to cause desorption of the analytes from active matrix sites. The presence of a modifier increases the solubility since the solvent is capable of interacting with the analytes with different types of interactions including dipole–dipole and hydrogen bonding [23, 24]. Also, the modifier enhances desorption of the analytes present in micro/nanopores in the matrix. Furthermore, it has been found that the presence of modifier in the ScCO_2_ in plant matrix samples causes swelling of the matrix which facilitates the solvent penetration and enhances the mass transfer of the analytes [25]. Rai et al. reported that the addition of 5 and 10% ethanol to ScCO_2_ (*v*:*v*) at 30 MPa increased the extracted amount of oil from MO seeds by 1.3 and 1.9 times, respectively [8]. Hence, in our study, the final optimal extraction condition was to add 1 mL ethanol and use 28 g of ScCO_2_ at 80 MPa and 57 °C.

The optimized UHPSFE method was compared with a conventional method based on sonication treatment in heptane. To make the comparison fair, the solvent/sample ratio (40:1 *g*:*g*) was kept equivalent to the UHPSFE method. The obtained extracted amount from the sonication treatment was 251 ± 22 mg oil/g seed of MO. This amount represents about 63% of the amount obtained by neat ScCO_2_ at 80 MPa and 57 °C. The conventional sonication method takes 1 h of extraction plus about 20 min of filtration and solvent evaporation, whereas, when the SFE method was run at a flow rate of 2 mL/min, it takes about 20 min to complete the extraction without a need for a cleanup procedure. The developed SFE method at the optimal conditions was used to extract oil from MP seeds for comparison with MO. At the optimal conditions for SFE, the oil amount extracted from MP seeds was 583 ± 33 mg. Figure 2b summarizes the obtained amount of oil at different extraction conditions, using different extraction techniques and different moringa species.

#### Extraction selectivity

**Fig. 3:**
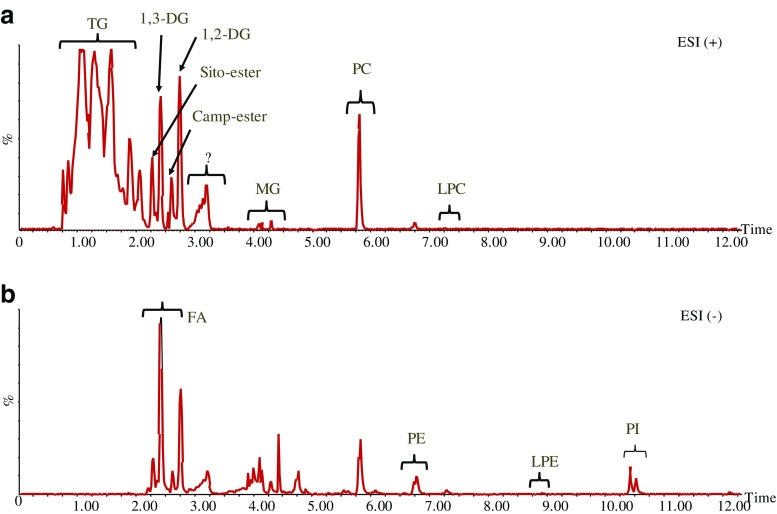
Examples of chromatograms obtained from the oil extracted from *Moringa peregrina* using the optimized extraction method. In **a** positive ESI and **b** negative ESI

**Fig. 4:**
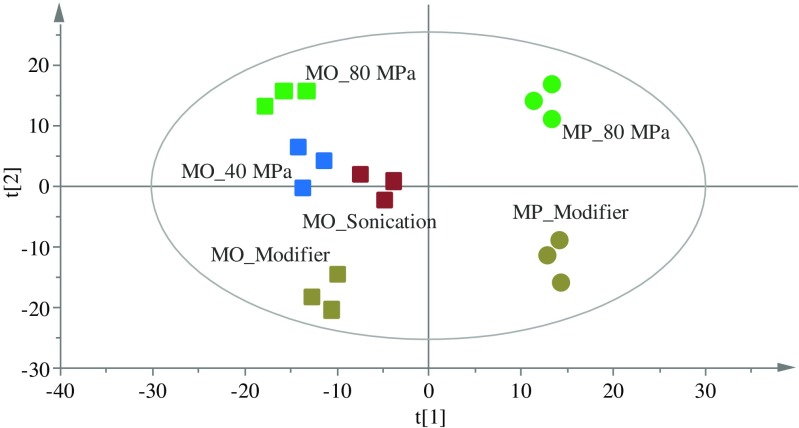
A PCA score plot of MS data of oil samples from two moringa species obtained under different extraction techniques and conditions

Extracted oil samples obtained using different methods were analyzed by UHPSFC/QTOF–MS. Screening the MS data obtained from both positive and negative ESI modes for pooled oils obtained at different extraction conditions from MO seeds showed that several lipid classes could be identified based on retention time, exact mass *m*/*z*, and MS/MS fragmentation patterns [17, 26]. These classes include sterol ester (SE), triacylglycerol (TG), diacylglycerol (DG), monoacylglycerol (MG), phosphatidylcholine (PC), lysophosphatidylcholine (LPC), phosphatidylethanolamine (PE), phosphatidylinositol (PI), phosphatidylglycerol (PG), and free fatty acids (FFAs). Typical chromatograms obtained from MP oil extracted at the optimum condition with ethanol are shown in Fig. 3. There is one lipid class detected at relatively high abundance eluted between 3.0 and 3.5 min which was not included in the discussion of the oil composition due to uncertainty in the identification. This class also has a feature of fatty acids as can be recognized by differences in *m*/*z* by 2 Da. In general, this class of lipid is detected uniquely in MP and might belong to glycolipids but this is not fully confirmed. Targeted peak picking for more than 300 lipid species (excluding this class) was followed to study the lipid composition and relative quantities of these species in the oil obtained at different extraction conditions (40 and 80 MPa), different extraction techniques (SFE and sonication), and two moringa species (MO and MP). The resulting peak areas were centered and scaled to unit variance and visualized by a PCA model (described variation, *R*^2^ = 0.91; predictive ability, *Q*^2^ = 0.77) as shown in Fig. 4. The first component described 66% of the total variation and the second component accounted for about 18%. The score plot shows a clear clustering between extraction methods and moringa species. The first component separates the data according to the origin of the moringa species while the second component separates the extraction method either as neat ScCO2 or in the presence of the modifier. The PCA model reveals that extraction selectivity varies with different pressures and presence of modifier. To understand the influence of these conditions on the selectivity, separated PCA models were calculated and fitted (see ESM, Figs. S2 and S3).

#### Effect of extraction pressure on the extracted oil composition

As mentioned above, the effect of pressure on the total amount of extracted oil is clear. It was suggested that a higher pressure increased the dielectric constant of ScCO_2_ and its electron donation and accepting capacity that enables interaction with polar head groups [27]. The PCA model (ESM Fig. S2) and ESM Table S1 present the identified compounds from MO oil extracted under 40 and 80 MPa at a fixed temperature (57 °C). The score plot of the PCA shows the clustering of the seed samples based on the extraction pressure. The loading plot indicates that most of the lipid species are extracted in relatively higher amount under higher pressure. The main detected components in the oil are TGs, DGs, and SEs. The clustering of the data discloses that at high pressure (80 MPa), lipids with longer fatty acid chains and a higher degree of unsaturation are extracted in higher relative abundance in comparison with lower pressure (40 MPa). Interestingly, some polar lipid species were detected uniquely in the oil extracted at 80 MPa compared to 40 MPa. This can be attributed to the change in the electron donating and accepting capacity of ScCO_2_ at high pressure as we mentioned earlier. However, detection of some of the phospholipid species using neat ScCO_2_ is an indication of the presence of phospholipids in significant amount in MO seeds. In order to investigate the polar lipids, oil extracted at 80 MPa in the presence of ethanol was analyzed and compared to neat ScCO_2_.

#### Effect of modifier

The most significant aspect of the addition of the modifier is that it increases the solubility range of the solvent to include more polar lipids. Although only 1 mL of the modifier (ethanol) was added to the sample after being placed in the extraction vessel, it was adequate to increase the extracted amount of the oil significantly compared to neat ScCO_2_ (Fig. 2b). The UHPSFC/QTOF–MS analysis and the PCA in Fig. S3 (see ESM) show that the modifier enhanced extraction of polar lipids to include phosphatidylinositol (PI) and phosphatidylserine (PS) (ESM Table S2). This could be explained by the increase of dipole–dipole and hydrogen bond interactions between ethanol and the oil components with polar function groups [28]. Table S2 (see ESM) shows phospholipid species detected higher under the addition of ethanol as a modifier. Another remark from the addition of the modifier is that lipids from TGs, DGs, and FAs with a higher degree of unsaturation are extracted significantly more under the addition of ethanol. This finding is in agreement with our finding in the separation of lipids by SFC [17], where lipids with a higher degree of saturation elute later with a higher percentage of organic co-solvent. This indicates that fatty acids with a higher degree of unsaturation have higher solubility in more polar solvents compared to neat ScCO_2_.

#### UHPSFE vs. sonication

Sonication treatment is considered as an efficient way to enhance the recovery of bioactive compounds from plant matrix [29]. As mentioned earlier in the extraction optimization section, sonication-assisted extraction gives lower recovery than UHPSFE. A PCA plot (ESM Fig. S4) was calculated for the data obtained from UHPSFE using neat ScCO_2_ and sonication extracts and illustrates differences in the selectivity. The data reveals that there are some unique lipid species extracted under either sonication or UHPSFE. In general, polar lipids that belong to phospholipids are extracted in higher amount with sonication compared to UHPSFE. Several species like PC 42:2, LPC 22:1, FA 22:4, PE 38:2, PI 32:2, and MG 22:6 are extracted uniquely under sonication. This can be attributed to the effect of the organic solvent which is swelling of the particles and the impact of sonication which is destruction of the cell wall and therefore better contact and interactions of the solvent with the particles. Other species of lipids are extracted at different ratios between UHPSFE and sonication without noticeable trends. The results also revealed that UPHSFE is more selective toward campesterol and stigmasterol esters than sonication. Studies reported no differences in the fatty acid composition of oil obtained by SFE or sonication [30, 31]. Thus, UHPSFE using neat ScCO_2_ might increase the extracted amount of oil but has a minor influence on the composition of the oil and fatty acid profiling compared to sonication-assisted extraction.

#### Oil composition in seeds of MO and MP

**Fig. 5:**
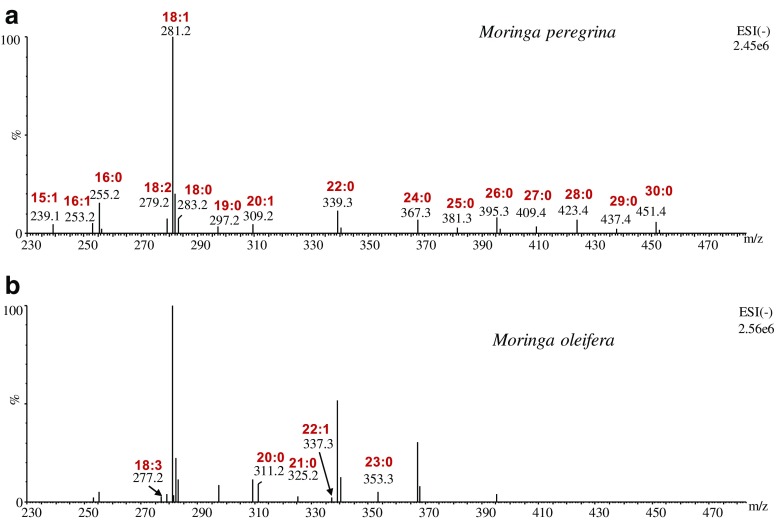
Extracted masses eluted between 2 and 3 min in UHPSFC/QTOF–MS in (−) ESI in oil samples from MP (**a**) and MO (**b**)

As a final comparison in this study, the oil compositions obtained using the developed UHPSFE method with modifier in MO and MP seeds were compared using the UHPSFC/QTOF–MS analysis method (ESM, Table S3 and Fig. S5). Figures S6 and S7 in the ESM illustrate the chromatograms for the oil from MO and MP in both (+/−) ESI-QTOF, respectively. Although MO has a lower total content of oil than MP, its composition is rich in phospholipids. For example, many species of LPC, PC, and LPE are detected uniquely in MO. In addition, most campesterol and stigmasterol esters are significantly higher in MO than in MP. However, cholesterol esters are more abundant in MP. Fahad et al. reported previously that cholesterol is not detected in MO while stigmasterol is significantly higher than in MP [32]. On the other hand, lipids with a higher degree of unsaturation are more pronounced in MP than in MO. Figure 5 shows detected masses in MP and MO in ESI (−) between retention times of 2 and 3 min, where free fatty acid elution is expected. MP is a rich source of very-long-chain fatty acids (C>22) such as lignoceric acid (C24), montanic acid (C28), and melissic acid (C30). For short-chain fatty acids, both species have similar profiling although some species are detected only in MO like heneicosylic acid (C21). From these results, we can observe the diversity of the lipid compositions in the same plant species. The differences in lipid composition are dependent not only on moringa species but also on other factors related to geographic origin, nutrients, and other growth conditions.

## Conclusion

An extraction method based on UHPSFE was developed to obtain oil from moringa seeds. Response surface methodology was used to optimize the operation conditions of pressure and temperature. Pressure was found to be the most important factor influencing the oil extractability. Extraction at 80 MPa and 57 °C was the optimal condition to achieve the highest extracted amount in the investigated range. Extraction at 80 MPa increased the extracted amount to 396 mg/g MO seeds compared to 278 mg/g MO seeds at 40 MPa. The presence of a modifier, ethanol, increased the extracted amount and also increased the abundance of polar lipids, mainly phospholipids, in the extracted oil. The results showed that sonication extraction is not as efficient to extract the oil from the seeds as the novel UHPSFE method. UHPSFC analysis revealed that moringa oil is composed of TGs, DGs, MGs, campesterol and stigmasterol ester, FAs, PCs, LPCs, PEs, LPEs, PIs, and PSs. MP has a higher content of oil than MO seeds. Phospholipids are more abundant in MO while MP has uniquely very-long-chain fatty acids.

## Electronic supplementary material

ESM 1 (PDF 387 kb)
