# Supplementary material for: Ultrahigh-pressure supercritical fluid extraction and chromatography of Moringa oleifera and Moringa peregrina seed lipids
Source: Anal Bioanal Chem. 2019 May 4;411(16):3685–93. doi: 10.1007/s00216-019-01850-x (PMC6571088; doi:10.1007/s00216-019-01850-x)
Supplement: Supplementary file 1 — (PDF 387 kb) [file 216_2019_1850_MOESM1_ESM.pdf]

Analytical and Bioanalytical Chemistry

Electronic Supplementary Material

Ultrahigh pressure supercritical fluid extraction and chromatography of *Moringa oleifera* and *Moringa peregrina* seed lipids

Yannick Nuapia Belo, Said Al-Hamimi, Luke Chimuka, Charlotta Turner

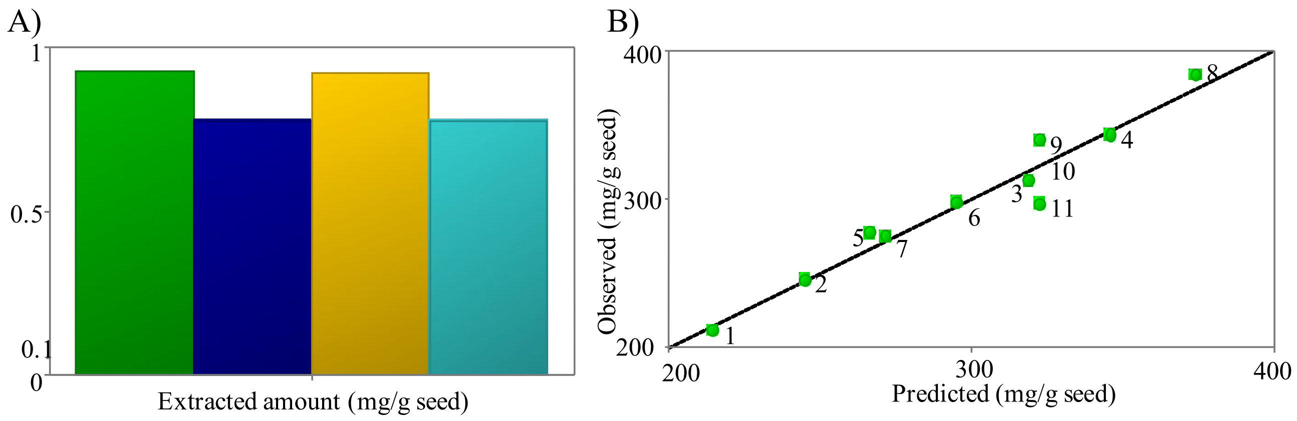

Fig. S1 (A) DOE model fitting shows the R^2 (94%) and Q^2 (85%) values with validity and repeatability evaluations. (B) The plot validates the model by plotting the predicted values with experimental values which resulted in linear relationship

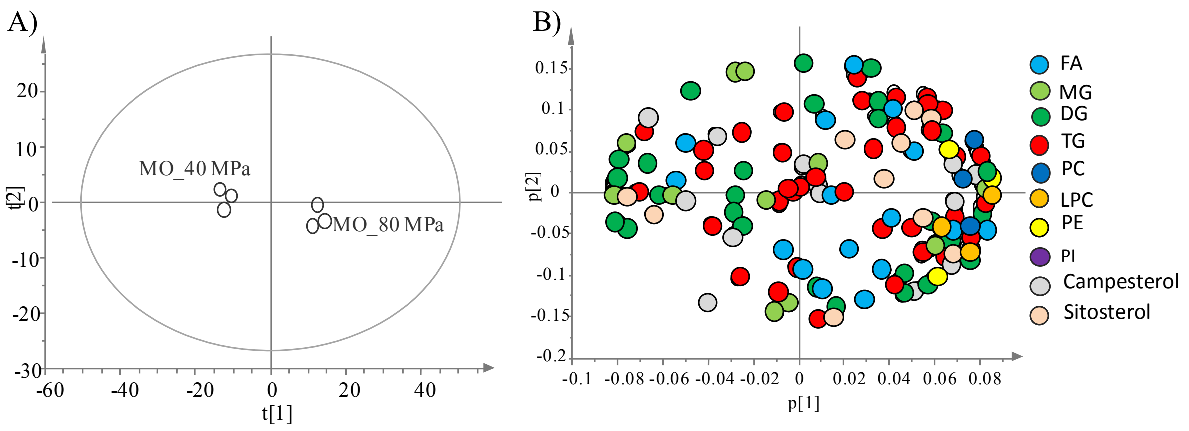

Fig. S2 PCA model shows the discrimination in the MS data (peak area) obtained at 40 and 80 MPa in both modes (+ and -) from *Moringa oleifera*, (A) is the score plot and (B) is the loading plot. The model describes variation, R^2=0.93; predictive ability, Q^2=0.80. The first component described 72% of total variation and the second component accounted for 22%

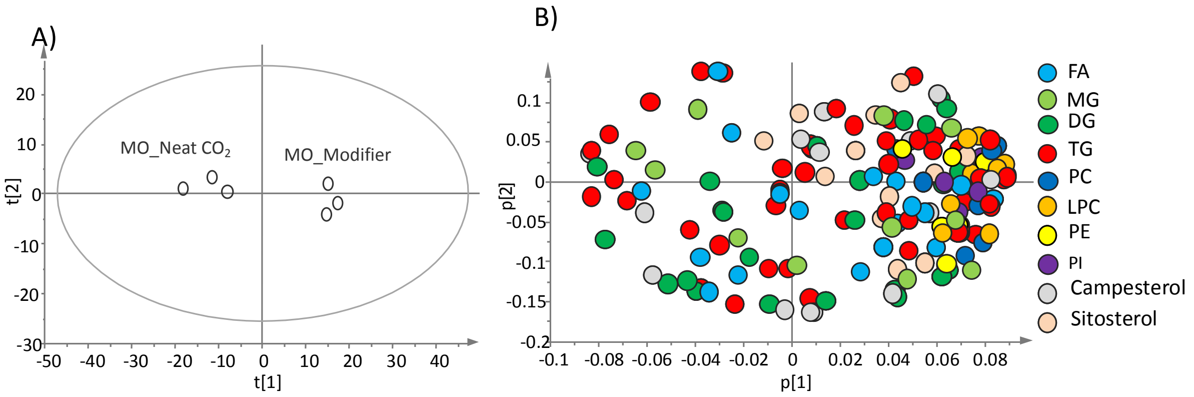

Fig. S3 PCA model showing the discrimination in the MS data (peak area) obtained from *Moringa oleifera* at the optimal condition of SFE with modifier and using neat CO_2 at 80 MPa. (A) is the score plot and (B) is the loading plot. The model describes variation, R^2=0.88; predictive ability, Q^2=0.79. The first component described 74% of total variation and the second component accounted for 16%

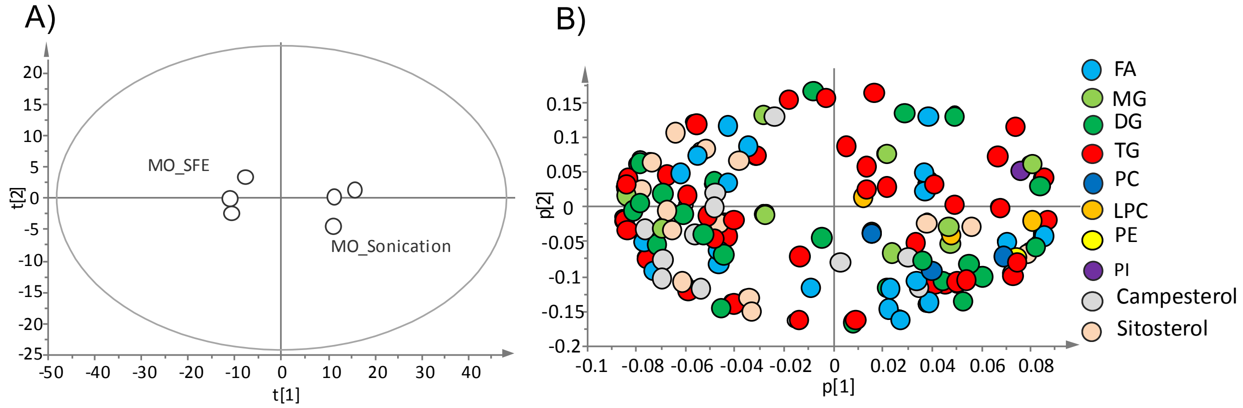

Fig. S4 PCA model shows the discrimination in the MS data (peak area) obtained from *Moringa oleifera* at the optimal condition of SFE and using conventional method (sonication). (A) is the score plot and (B) is the loading plot. The model describes variation, R^2=0.83; predictive ability, Q^2=0.76. The first component described 65% of total variation and the second component accounted for 25%

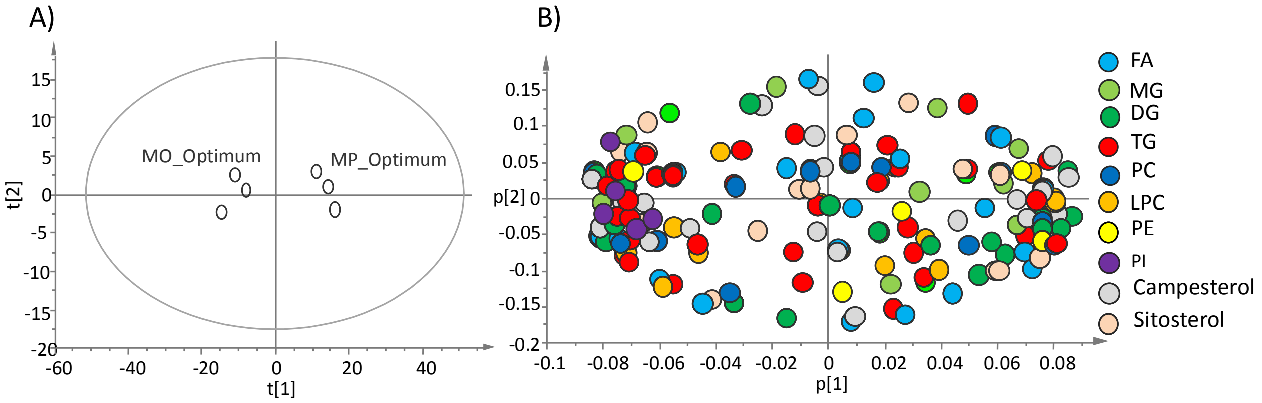

Fig. S5 PCA model shows the discrimination in the MS data (peak area) obtained from *Moringa oleifera* at the optimal condition of SFE with modifier and using neat CO_2 at 80 MPa. (A) is the score plot and (B) is the loading plot. The model describes variation, R^2=0.88; predictive ability, Q^2=0.79. The first component described 74% of total variation and the second component accounted for 16%

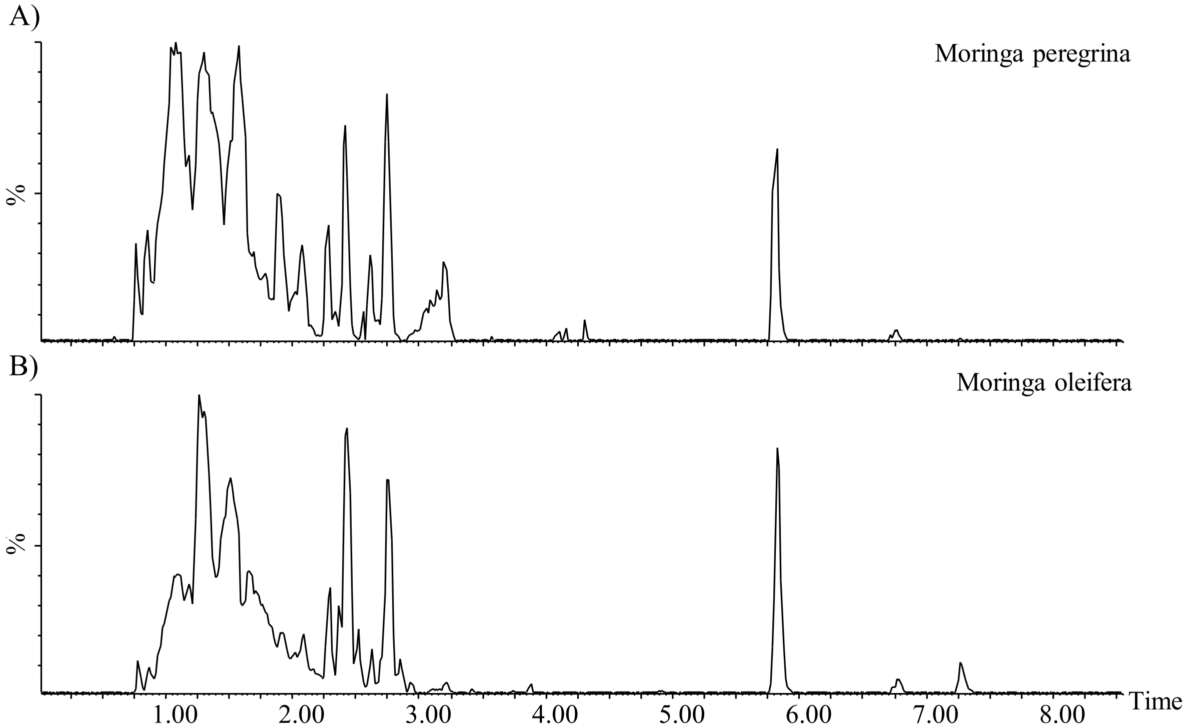

Fig. S6 The (BPI) chromatograms in positive ESI for *Moringa peregrina* (A) and *oleifera* (B)

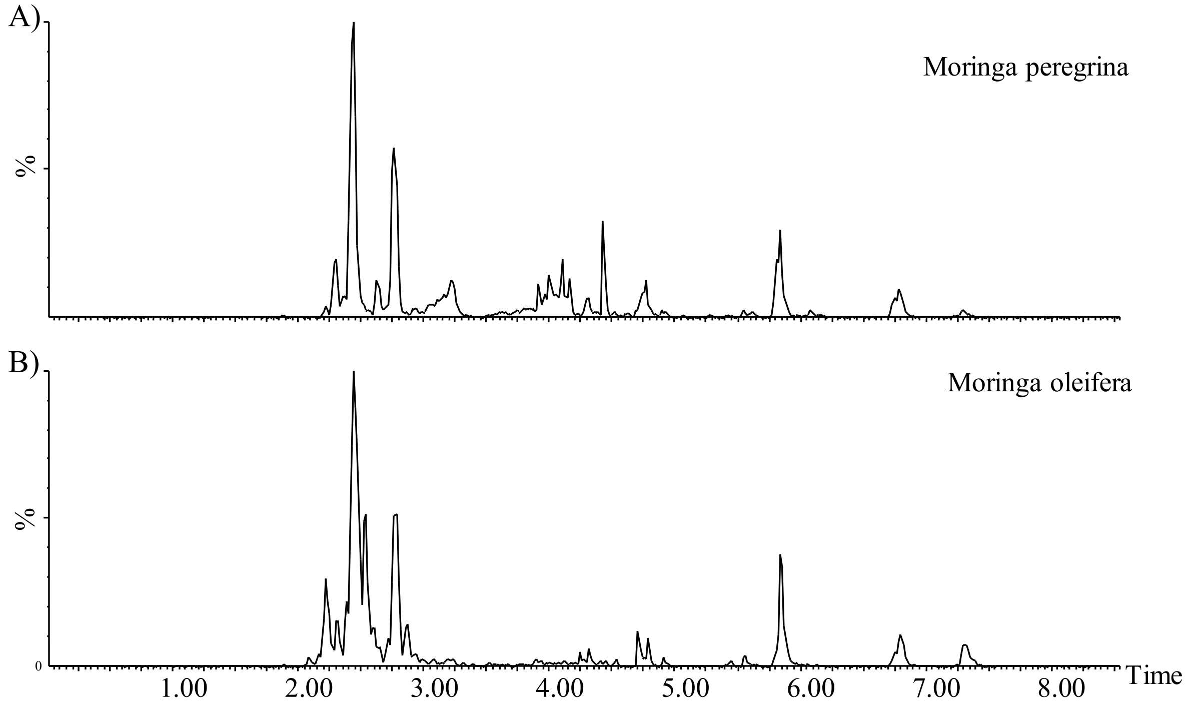

Fig. S7 The (BPI) chromatograms in negative ESI for *Moringa peregrina* (A) and *oleifera* (B)

Table S1 Relative peak area of lipids detected in oil extracted from *Moringa Oleifera* seeds at 40 and 80 MPa

**Lipid species detected higher at 80 MPa**		
**Identification**	**80 MPa**	**40 MPa**
TG 54:7	1±0.47	0.06±0.01
20:0-Campesterol	1±0.12	0.22±0.03
16:0-Campesterol	1±0.25	0.25±0.03
TG 42:0	1±0.26	0.27±0.03
1,2 DG 36:6	1±0.1	0.31±0.04
TG 55:5	1±0.25	0.35±0.04
TG 50:4	1±0.07	0.45±0.05
TG42:2	1±0.3	0.46±0.05
TG 44:5	1±0.26	0.47±0.06
TG 60:3	1±0.03	0.52±0.06
TG 58:2	1±0.06	0.52±0.06
TG 55:4	1±0.07	0.52±0.06
TG 49:3	1±0.14	0.52±0.06
18:3-Campesterol	1±0.1	0.55±0.06
TG 58:3	1±0.03	0.56±0.07
TG 53:3	1±0.07	0.56±0.07
TG 52:4	1±0.05	0.57±0.07
TG 49:1	1±0.06	0.57±0.07

**Identification**	**80 MPa**	**40 MPa**
TG 50:0	1±0.09	0.67±0.08
TG 54:5	1±0.1	0.67±0.08
TG 50:1	1±0.1	0.67±0.08
TG 48:0	1±0.1	0.67±0.08
TG 56:0	1±0.14	0.67±0.08
TG 53:6	1±0.06	0.68±0.08
TG 48:1	1±0.05	0.69±0.08
TG 51:3	1±0.08	0.69±0.08
TG 55:2	1±0.1	0.69±0.08
TG 53:2	1±0.03	0.7±0.08
TG 54:3	1±0.04	0.7±0.08
TG 51:5	1±0.08	0.7±0.08
TG 60:5	1±0.26	0.71±0.2
1,3 DG 34:4	1±0.02	0.71±0.2
1,3 DG 35:5	1±0.11	0.72±0.25
TG 50:3	1±0.07	0.72±0.08
1,2 DG 35:6	1±0.07	0.72±0.08
TG 54:2	1±0.05	0.74±0.09
TG 58:4	1±0.11	0.74±0.09

TG 44:2	1±0.22	0.57±0.07
TG 48:3	1±0.07	0.58±0.07
TG 42:1	1±0.25	0.58±0.07
TG 46:5	1±0.23	0.59±0.07
TG 60:2	1±0.04	0.6±0.07
TG 51:3	1±0.05	0.6±0.07
TG 49:2	1±0.07	0.6±0.07
TG 53:4	1±0.08	0.6±0.07
TG 49:0	1±0.12	0.61±0.07
TG 56:5	1±0.09	0.62±0.07
TG 56:4	1±0.07	0.63±0.07
TG 51:2	1±0.08	0.63±0.07
TG 46:1	1±0.21	0.63±0.07
TG 53:5	1±0.04	0.64±0.07
TG 60:3	1±0.16	0.64±0.07
1,2 DG 35:4	1±0.02	0.65±0.08
TG 54:6	1±0.08	0.66±0.08
TG 46:2	1±0.15	0.66±0.08
TG 48:2	1±0.06	0.67±0.08

TG 55:6	1±0.02	0.75±0.09
TG 52:3	1±0.07	0.75±0.09
TG 56:1	1±0.06	0.75±0.09
TG 52:0	1±0.12	0.75±0.09
TG 56:3	1±0.12	0.76±0.09
TG 54:4	1±0.08	0.78±0.09
TG 54:1	1±0.1	0.78±0.09
1,2 DG 35:3	1±0.02	0.79±0.09

**Lipid species detected higher at 40 MPa**		
1,3 DG 38:1	1±0.03	1.24±0.14
1,3 DG 34:3	1±0.14	1.45±0.17
MG 20:4	1±0.04	1.46±0.17
1,2 DG 35:0	1±0.23	1.47±0.17
TG 55:3	1±0.07	1.69±0.2
1,2 DG 32:3	1±0.1	1.7±0.2
TG 60:5	1±0.26	1.7±0.2
1,3 DG 34:4	1±0.02	1.71±0.2
1,2 DG 38:4	1±0.08	1.71±0.2
1,3 DG 32:3	1±0.1	1.71±0.2
MG 18:0	1±0.19	1.79±0.21
1,3 DG 35:5	1±0.11	2.17±0.25
MG 18:1	1±0.1,2	2.28±0.27
MG 16:0	1±0.87	3.32±0.39

**Lipid species detected uniquely at 80 MPa**
PC 34:1
PC 32:2
LPC 16:0
PC 36:1
PE 38:2
PE 36:2
PE 34:1

Table S2 Relative peak area of lipid species detected in the oil obtained from *Moringa oleifera* seeds using neat CO_2 and CO_2 with modifier (ethanol) at 80 MPa

Lipid species detected higher at 80 MPa with modifier (ethanol)		
**Identification**	**Modifier**	**neat CO_2**
20:2-Stigmasterol	1±0.03	0.01±0
FA 16:1	1±0.33	0.01±0.01
22:2-Sitosterol	1±0.03	0.01±0.01
20:3-Campesterol	1±0.1	0.01±0.02
1,3 DG 36:6	1±0.02	0.02±0
TG 50:4	1±0.03	0.02±0
1,3DG 32:2	1±0.03	0.02±0
20:0-Campesterol	1±0.03	0.02±0
TG 58:5	1±0.03	0.02±0
FA 18:3	1±0.15	0.02±0.02
TG 50:5	1±0.36	0.02±0.04
18:3-Sitosterol	1±0.04	0.03±0
TG 53:3	1±0.12	0.03±0
20:2-Sitosterol	1±0.06	0.03±0
20:1-Stigmasterol	1±0.04	0.03±0
MG 18:2	1±0.05	0.04±0.03
FA 20:1	1±0.23	0.04±0.04

**Identification**	**Modifier**	**neat CO_2**
20:2-Campesterol	1±0.03	0.1,2±0.04
FA 20:0	1±0.14	0.1,2±0.15
TG 60:2	1±0.06	0.1,3±0
1,2 DG 38:3	1±0.04	0.14±0.03
1,2 DG 35:0	1±0.08	0.16±0.04
TG 44:3	1±0.49	0.17±0.08
20:3-Sitosterol	1±0.06	0.19±0.01
18:3-Campesterol	1±0.11	0.21±0.02
16:1-Sitosterol	1±0.03	0.22±0.01
1,3 DG 36:5	1±0.05	0.23±0.02
16:3-Sitosterol	1±0.04	0.23±0.06
20:0-Sitosterol	1±0.03	0.24±0.03
1,3 DG 34:4	1±0.07	0.25±0
20:1-Sitosterol	1±0.05	0.25±0.03
22:0-Campesterol	1±0.11	0.25±0.08
16:0-Sitosterol	1±0.02	0.27±0.02
1,2 DG 32:3	1±0.01	0.29±0.03

16:1-Campesterol	1±0	0.05±0.02
1,3 DG 36:7	1±0.02	0.05±0.06
FA 22:4	1±0.31	0.05±0.09
18:2-Stigmasterol	1±0.06	0.06±0
18:1-Stigmasterol	1±0.05	0.06±0.01
22:1-Stigmasterol	1±0.18	0.07±0.03
16:2-Campesterol	1±0.01	0.08±0
22:0-Sitosterol	1±0.08	0.08±0.01
16:2-Sitosterol	1±0.02	0.08±0.01
22:1-Sitosterol	1±0.04	0.08±0.01
20:1-Campesterol	1±0.04	0.08±0.01
TG 60:4	1±0.12	0.08±0.02
16:3-Campesterol	1±0.04	0.09±0.01
1,3 DG 36:3	1±0.02	0.09±0.01
TG 60:3	1±0.32	0.09±0.01
22:2-Campesterol	1±0.04	0.1±0.01
18:2-Sitosterol	1±0.01	0.1±0.01
16:2-Stigmasterol	1±0.01	0.1±0.02
1,3 DG 38:3	1±0.01	0.1±0.02
FA 18:0	1±0.18	0.11±0.09
1,3 DG 32:3	1±0.01	0.29±0.03
16:3-Stigmasterol	1±0.04	0.33±0.03
1,3 DG 34:3	1±0.01	0.34±0.05
1,3 DG 38:4	1±0.02	0.39±0.03
1,3 DG 35:5	1±0.04	0.4±0.05
16:0-Campesterol	1±0.09	0.41±0.1
18:2-Campesterol	1±0.06	0.44±0.03
18:3-Stigmasterol	1±0.11	0.45±0.08
1,3 DG 32:0	1±0.07	0.45±0.29
1,2 DG 38:0	1±0	0.51±0.05
1,3 DG 35:6	1±0.03	0.57±0.04
1,2 DG 38:1	1±0	0.75±0.03
1,2 DG 32:0	1±0.07	0.75±0.1,2

**Lipid species detected uniquely at 80 MPa with modifier (ethanol)**		
22:2-Stigmasterol	20:3-Campesterol	PI 36:5
22:3-Sitosterol	LPC 22:1	LPC 20:3
PC 34:0	PC 35:3	LPC 20:2
PC 42:2	PC 37:3	18:0-Campesterol
MG 22:6	18:1-Campesterol	LPC 16:1
PC 35:1	PC 36:1	LPC 18:0
PC 36:1	FA 18:2	LPC 18:1
PE 36:2	22:3-lesterol	LPC 16:2
PC 34:1	PC 35:4	LPC 18:3
PC 32:2	22:3-Campesterol	PC 34:5
PE 38:2	PC 37:2	PE 34:0
PE 34:1	LPC 18:2	FA 22:3
LPC 19:0	PE 38:2	PE 32:1
PE 36:2	FA 20:2	LPE 17:0
FA 22:1	LPC 19:1	PC 35:5
LPC 20:5	PI 36:0	PC 40:2

Table S3 Relative peak area of lipid species detected in the oil obtained from *Moringa Oleifera* (MO) and *Moringa Peregrina* (MP) seeds using neat CO_2 with modifier (ethanol) at 80 MPa

**Lipid species detected higher in MP**		
**Identification**	**MP**	**MO**
16:0-Campesterol	1±0.34	0.02±0.02
22:1-Stigmasterol	1±0.04	0.04±0
22:2-Sitosterol	1±0.04	0.04±0
22:0-Campesterol	1±0.58	0.04±0.02
20:1-Stigmasterol	1±0.03	0.05±0
20:2-Sitosterol	1±0.03	0.05±0
MG 20:4	1±0	0.05±0.01
20:0-Sitosterol	1±0.08	0.06±0
18:3-Stigmasterol	1±0.03	0.06±0
MG 18:1	1±0.04	0.06±0.01
22:0-Stigmasterol	1±0.18	0.06±0.01
22:1-Sitosterol	1±0.18	0.06±0.01
1,3 DG 38:4	1±0.02	0.07±0
16:3-Stigmasterol	1±0.03	0.07±0
18:0-Stigmasterol	1±0.05	0.08±0
18:1-Sitosterol	1±0.05	0.08±0
20:0-Stigmasterol	1±0.04	0.08±0.01
20:1-Sitosterol	1±0.04	0.08±0.01

**Identification**	**MP**	**MO**
TG 60:3	1±0.03	0.47±0.01
TG 60:2	1±0.01	0.48±0.02
TG 52:5	1±0.11	0.48±0.03
TG 60:6	1±0.18	0.48±0.05
1,3 DG 35:5	1±0.04	0.49±0.01
1,3 DG 36:0	1±0.02	0.49±0.02
20:2-Campesterol	1±0.03	0.49±0.04
PE 36:2	1±0.09	0.49±0.09
PE 34:2	1±0.25	0.5±0.1
TG 60:5	1±0.08	0.5±0.11
PC 36:3	1±0.01	0.51±0.02
PC 33:2	1±0.01	0.52±0.04
PE 36:1	1±0.04	0.52±0.11
18:2-Sitosterol	1±0.06	0.54±0.12
18:1-Stigmasterol	1±0.06	0.54±0.12
20:3-Stigmasterol	1±0.11	0.55±0.02
1,2 DG 32:3	1±0.01	0.56±0.01
1,2 DG 35:4	1±0.01	0.56±0.02

18:0-Sitosterol	1±0.03	0.09±0	LPE 16:0	1±0.07	0.56±0.06
LPC 20:4	1±0.02	0.1±0.01	PC 35:5	1±0.02	0.58±0.06
1,3 DG 38:0	1±0.04	0.12±0.01	TG 54:0	1±0.04	0.59±0.1,3
1,2 DG 38:0	1±0.04	0.12±0.01	PC 37:1	1±0.01	0.6±0.02
16:0-Sitosterol	1±0	0.13±0	TG 58:3	1±0	0.6±0.02
LPE 18:1	1±0.01	0.14±0.01	PC 36:1	1±0.01	0.6±0.03
1,2 DG 36:4	1±0.05	0.14±0.01	PC 36:2	1±0.03	0.6±0.04
PC 40:1	1±0.11	0.14±0.14	16:2-Campesterol	1±0.02	0.62±0.01
LPC 18:1	1±0.01	0.16±0	PC 36:0	1±0.01	0.64±0.02
LPC 20:1	1±0.04	0.17±0	1,3 DG 36:1	1±0.03	0.67±0.01
16:0-Stigmasterol	1±0	0.18±0.01	TG 54:1	1±0.05	0.68±0.01
16:1-Sitosterol	1±0	0.18±0.01	PC 35:4	1±0.02	0.68±0.02
1,3 DG 38:1	1±0	0.19±0.01	PC 34:3	1±0.02	0.71±0.02
1,2 DG 38:1	1±0	0.19±0.01	PC 37:2	1±0.02	0.72±0.07
PE 34:1	1±0.46	0.21±0.02	PC 38:0	1±0.02	0.73±0.02
1,2 DG 34:3	1±0.01	0.21±0.05	PC 38:3	1±0.05	0.74±0.1
1,2 DG 38:4	1±0.14	0.22±0.02	PC 35:1	1±0	0.75±0.03
20:2-Stigmasterol	1±0.06	0.22±0.06	16:2-Stigmasterol	1±0.04	0.77±0.02
20:3-Sitosterol	1±0.06	0.22±0.06	16:3-Sitosterol	1±0.04	0.77±0.02
20:1-Campesterol	1±0.06	0.26±0	1,3 DG 38:3	1±0.04	0.77±0.02
LPC 18:0	1±0.1	0.26±0.01	TG 55:6	1±0.08	0.79±0.08
PC 36:4	1±0.03	0.26±0.02	PE 38:5	1±0.1	0.81±0.03

PC 42:1	1±0.06	0.26±0.26
18:3-Sitosterol	1±0.11	0.27±0
18:2-Stigmasterol	1±0.11	0.27±0
18:3-Campesterol	1±0.16	0.27±0.01
1,2 DG 34:4	1±0.06	0.28±0
1,3 DG 34:4	1±0.06	0.29±0
22:2-Campesterol	1±0.29	0.34±0.01
LPC 16:1	1±0.05	0.35±0.27
PC 40:4	1±0.18	0.35±0.35
PC 38:2	1±0.02	0.36±0.01
TG 60:4	1±0.02	0.37±0.02
18:0-Campesterol	1±0.02	0.37±0.04
18:2-Campesterol	1±0.01	0.37±0.18
TG 54:5	1±0.06	0.39±0.02
16:2-Sitosterol	1±0.01	0.39±0.1
TG 58:4	1±0.02	0.47±0

TG 54:2	1±0.08	0.81±0.06
TG 52:0	1±0.03	0.83±0.02
TG 56:3	1±0.05	0.85±0.03
TG 53:3	1±0.06	0.86±0
1,3 DG 35:2	1±0.04	0.86±0.01
TG 54:4	1±0.04	0.89±0.04
1,2 DG 38:3	1±0.02	0.91±0.02
TG 56:2	1±0.03	0.91±0.02
16:1-Stigmasterol	1±0.01	0.39±0.1
1,3 DG 38:2	1±0.01	0.39±0.1
TG 58:5	1±0.02	0.43±0.03
PC 38:1	1±0.02	0.44±0.01
TG 58:2	1±0.04	0.45±0.01
1,2 DG 38:2	1±0.01	0.45±0.11
PE 32:0	1±0.1	0.45±0.22
TG 56:5	1±0.06	0.46±0.11

Lipid species detected higher in MO		
Identification	MP	MO
TG 50:4	1±0.03	1.11±0.01
TG 56:4	1±0.01	1.11±0.06
PC 33:4	1±0.05	1.1,2±0.04
PC 35:0	1±0.02	1.1,3±0.05
1,2 DG 35:5	1±0.04	1.14±0.06
TG 53:6	1±0.03	1.17±0.01
TG 52:4	1±0.03	1.18±0.04
TG 51:3	1±0.04	1.24±0.03
TG 52:2	1±0.04	1.28±0.02
1,3 DG 32:0	1±0.07	1.35±0
TG 54:3	1±0.08	1.38±0.1
TG 54:6	1±0.01	1.42±0.21
PC 34:0	1±0.01	1.43±0.05
PC 34:1	1±0.03	1.46±0.05
PC 35:3	1±0.05	1.47±0.19
1,3 DG 34:0	1±0.05	1.51±0.07
PC 34:2	1±0.02	1.53±0.04
TG 49:2	1±0.04	1.53±0.07
PC 33:5	1±0.08	1.54±0.31

Identification	MP	MO
CE 20:1	1±0.2	2.27±0.14
TG 46:0	1±0.04	2.15±0.06
TG 50:2	1±0.04	2.22±0.07
PC 32:2	1±0.05	2.26±0.08
PE 38:1	1±0.38	2.26±0.18
TG 50:0	1±0.03	2.39±0.1
TG 50:1	1±0.03	2.42±0.11
PC 33:1	1±0.08	2.51±0.2
PC 32:0	1±0	2.52±0.16
TG 48:2	1±0.03	2.59±0.07
TG 53:1	1±0.01	2.75±0.07
CE16:0	1±0.11	2.76±0.04
TG42:1	1±0.1,2	2.82±0
TG 49:0	1±0.15	2.85±0.12
TG42:2	1±0.05	2.88±0.07
1,3 DG	1±0.18	2±0.49
CE 18:3	1±0.14	3.03±0.04
TG 50:3	1±0.04	3.11±0.05
TG 49:1	1±0.33	3.24±0.03

TG 51:5	1±0.03	1.55±0.07
PI 36:2	1±0.02	1.71±0.01
PI 36:1	1±0.19	1.71±0.11
1,2 DG 34:2	1±0.05	1.72±0.02
TG 52:3	1±0.05	1.75±0
TG 55:4	1±0.02	1.75±0.09
1,3 DG 34:1	1±0.04	1.77±0.07
TG 49:3	1±0.03	1.78±0.15
1,2 DG 32:1	1±0.04	1.81±0.04
PE 35:1	1±0.05	1.81±0.43
TG 56:6	1±0.01	1.84±0.07
1,2 DG 35:6	1±0.09	1.86±0.08
TG 46:2	1±0.02	1.87±0.05
PI 36:0	1±0.03	1.93±0.23
TG 55:2	1±0.04	1.96±0.01
PC 33:3	1±0.04	1.96±0.41
TG 46:1	1±0.02	1.97±0.02
TG 50:5	1±0.2	2.05±0.42
PC 32:1	1±0.01	2.08±0.08

1,3DG 32:2	1±0.09	3.25±0.15
TG 48:1	1±0.03	3.32±0.04
CE 16:3	1±0.01	3.38± 0.52
TG 42:0	1±0.01	3.42±0.02
TG 55:5	1±0.04	3.42±0.02
TG 51:1	1±0.01	3.45±0.08
1,2 DG 36:5	1±0.01	3.52±0.16
TG 44:0	1±0.1,3	3.91±0.03
TG 48:0	1±0.03	4.32±0.05
PI 36:4	1±0.03	4.33±0.02
TG 48:3	1±0.05	4.49±0.2
TG 53:2	1±0.02	4.89±0.14
CE 19:0	1±0.19	4.95±1.36
TG 44:2	1±0.03	4.96±0.06
PI 34:2	1±0.15	5.52±0.26
PI 34:1	1±0.03	5.66±0.25
PI 34:0	1±0.06	5.74±0.02
CE 18:2	1±1	5.84±1.09
PI 36:3	1±1	6.82±0.57
TG 44:3	1±0.03	6.9±0.21

**Species detected uniquely in MP**	**Species detected uniquely in MO**	
TG 51:4	MG 18:0	MG 22:6
TG 53:0	LPC 22:0	LPE 18:0
TG 51:0	PC 40:2	LPC 18:3
1,3 DG 35:4	LPC 19:0	22:0-Sitosterol
1,3 DG 36:5	PC 36:6	1,3 DG 36:6
TG 51:2	TG 52:6	TG 51:4
MG 20:5	PC 38:8	1,3 DG 35:4
TG 52:6	PC 34:4	TG 51:0
	LPE 18:2	TG 51:2
	PC 36:5	1,3 DG 36:5
	PC 38:7	TG 53:0
